# Incidentally Discovered Aortic Thrombosis in a Patient Undergoing Capecitabine and Oxaliplatin Chemotherapy for Colon Cancer

**DOI:** 10.7759/cureus.39042

**Published:** 2023-05-15

**Authors:** Kyle A Burton, Steven Gualdoni, Sheetal Acharya

**Affiliations:** 1 Medicine, Upper Peninsula Health System, Marquette, USA; 2 Medicine, Michigan State University College of Human Medicine, East Lansing, USA; 3 Hematology, Upper Peninsula Health System, Marquette, USA

**Keywords:** chemotherapy, aortic thrombosis, oxaliplatin, capecitabine, colon cancer

## Abstract

This case report describes a 68-year-old male who presented to the emergency department (ED) with nausea, vomiting, abdominal pain, diarrhea, and fatigue after starting adjuvant combination chemotherapy with capecitabine and oxaliplatin two weeks prior. Further evaluation of this patient in the ED revealed an incidentally discovered aortic thrombosis, of which this patient did not exhibit any specific symptoms. This case, among a few others, has described the development of arterial thrombosis in patients with cancer undergoing combination chemotherapy with capecitabine and oxaliplatin.

## Introduction

Hypercoagulability in the setting of cancer is a common occurrence that results from a multitude of pathologic mechanisms that predispose cancer patients to the development of thrombosis [1]. Venous thromboembolism is the most frequently observed type of thrombosis in cancer patients [2]. Arterial thrombosis is much less prevalent than venous thrombosis in cancer patients, but arterial thrombosis, including aortic thrombosis, can also pose a life-threatening risk. Chemotherapeutic drugs, including platinum-based agents, vascular endothelial growth factor inhibitors, tyrosine kinase inhibitors, and taxanes, have been associated with higher rates of arterial thromboembolism [3]. The management of arterial thrombosis of uncertain etiology often requires a review of the literature and often involves the input of hematologists [4]. Here, we present a case of a 68-year-old male with a history of stage IIIA colon cancer who presented to the emergency department with symptoms of nausea, vomiting, abdominal pain, diarrhea, and fatigue and received a CT scan of the abdomen and pelvis, which incidentally revealed a mural thrombus in the aortic wall, extending from the renal arteries to the mid-abdominal aorta. This case report highlights the importance of awareness of potential arterial thrombotic complications in patients undergoing chemotherapy, especially with combination chemotherapy of capecitabine and oxaliplatin.

## Case presentation

A 68-year-old Caucasian male with a past medical history of hypertension, obstructive sleep apnea, former cigarette smoking, chronic obstructive pulmonary disease (COPD), and stage IIIA colon cancer, who was later determined to be a carrier for autosomal recessive congenital dihydropyrimidine dehydrogenase (DPD) deficiency, presented to the emergency department (ED) with nausea, vomiting, abdominal pain, diarrhea, and fatigue. The patient was started on ciprofloxacin and metronidazole for concerns of infectious etiology. He also received intravenous (IV) ondansetron and morphine for nausea and pain control, respectively, at this time. The patient was not taking any anti-platelet or anti-coagulant medications on admission. Two weeks prior to his emergency department visit, this patient had undergone laparoscopic sigmoid colectomy for his stage IIIA colon cancer. His colon cancer involved a sigmoid lesion, with one of 16 lymph nodes involved, with the following staging characteristics: TX, N1a, M0, low-risk stage IIIa, and microsatellite instability (MSI) stable. The patient began adjuvant chemotherapy with capecitabine and oxaliplatin just two weeks prior to presenting to the ED, which began two months after his laparoscopic sigmoid colectomy.

**Figure 1:**
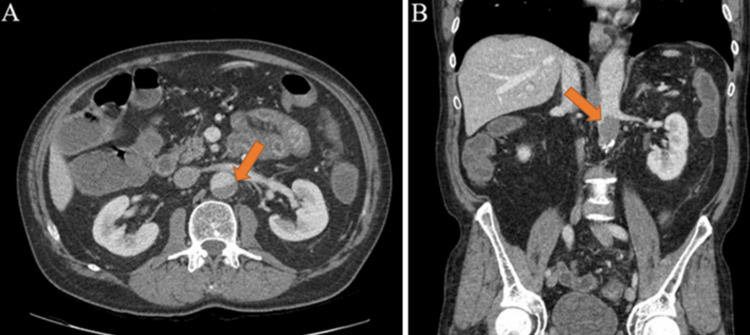
CT of the abdomen/pelvis showing aortic thrombosis (arrows) two weeks after beginning combination chemotherapy with capecitabine-oxaliplatin. A: Axial view. B: Sagittal view.

Significant laboratory findings at this time included a white blood cell count of 3,200 white blood cells per microliter, hemoglobin of 15.0 grams per deciliter, platelets of 241,000 platelets per microliter, and an absolute neutrophil count of 700 neutrophils per microliter. Given the patient’s acute onset of nausea, vomiting, and diarrhea, hematology was consulted as this patient’s clinical presentation was concerning for side effects resulting from his chemotherapy regimen. Given the patient’s symptoms and low absolute neutrophil count, inpatient admission was recommended and the patient was hospitalized for one week. In the ED, the patient underwent a CT scan of the abdomen and pelvis with IV contrast, which did not show any indications of an infectious etiology that could explain the patient's clinical symptoms. This CT scan revealed a new mural thrombus in the aortic wall with no change in aortic caliber. This concentric mural thrombus was present in the mid-abdominal aorta and extended from roughly the level of the renal arteries inferiorly to the mid-abdominal aorta, as shown in Figure 1.

**Figure 2:**
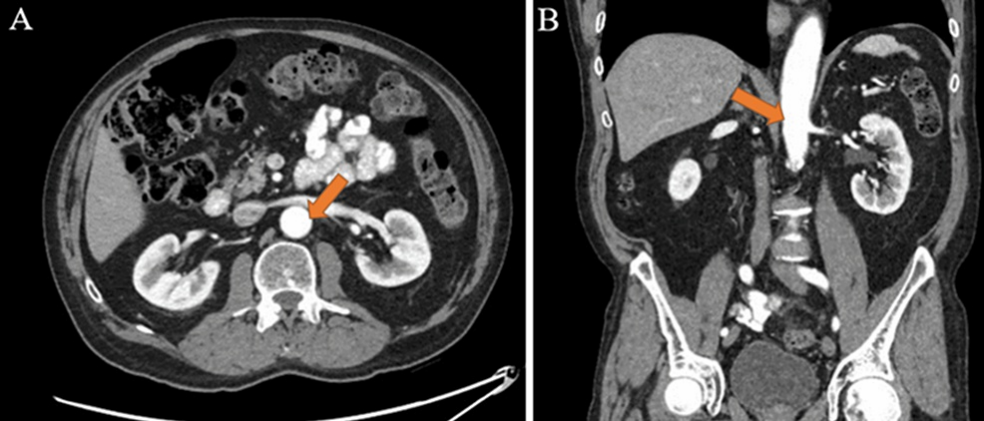
CT of the abdomen/pelvis with no aortic thrombosis present (as shown by the arrows) six weeks prior to the initiation of capecitabine-oxaliplatin chemotherapy. A: Axial view. B: Sagittal view.

The patient did not have any symptoms to suggest the presence of this incidentally discovered aortic thrombus. He was started on anticoagulation therapy with heparin and was transitioned to rivaroxaban on discharge, which he continued for six months. Due to the severity of the patient’s symptoms as well as the neutropenia noted on lab work, gene sequencing was ordered for DPD deficiency. This gene sequencing later revealed that the patient is a carrier for autosomal recessive congenital DPD deficiency. A deficiency of DPD may cause severe or potentially fatal toxicity in patients taking fluoropyrimidine drugs such as capecitabine [5]. Given this acute arterial thrombosis, the patient’s DPD deficiency carrier status, and the severity of the patient’s symptoms, the patient’s chemotherapy regimen was discontinued permanently. Shared decision-making with the patient determined that he would elect to proceed with active surveillance. Six weeks prior to presenting to the emergency department, the patient had received a CT scan of the abdomen/pelvis, which did not show any thrombus within the aortic wall, as shown in Figure 2. This CT imaging was obtained one month after the patient underwent laparoscopic sigmoid colectomy.

## Discussion

This report of an incidentally discovered aortic thrombosis in a patient undergoing capecitabine and oxaliplatin chemotherapy for stage IIIA colon cancer raises important considerations regarding the relationship between chemotherapy regimens and thrombotic events. Chemotherapy-induced venous thrombosis is a well-known complication of cancer treatment [2]. The development of arterial thrombosis has been associated with chemotherapeutic agents, including platinum-based agents, vascular endothelial growth factor inhibitors, tyrosine kinase inhibitors, and taxanes [3]. While chemotherapy-induced arterial thrombosis is less prevalent, there have been several reported cases, in addition to this case, implicating combination chemotherapy with capecitabine-oxaliplatin as a likely culprit.

One case report describes a patient with a resected adenocarcinoma of the sigmoid colon who developed an acute aortic thrombosis during adjuvant capecitabine-oxaliplatin chemotherapy [6]. Another case describes a patient who developed an aortic thrombosis while undergoing combination chemotherapy with capecitabine-oxaliplatin plus bevacizumab for the treatment of metastatic colon cancer [7]. This evidence suggests that patients who undergo chemotherapy using the combination of capecitabine and oxaliplatin may face a higher likelihood of developing arterial thrombosis.

Interestingly, the literature also reports arterial thrombotic events in patients receiving chemotherapy for cancers other than colon cancer. One case report describes a patient with metastatic gastric adenocarcinoma who developed an acute arterial thrombosis within the abdominal aorta and lumbar and right common iliac artery shortly after starting combination chemotherapy with epirubicin, oxaliplatin, and capecitabine [8]. This suggests that the risk of arterial thrombosis may extend beyond colon cancer and should be considered in patients with various malignancies undergoing chemotherapy with capecitabine and oxaliplatin, among other regimens. Additionally, capecitabine has been associated with various cardiovascular adverse effects [9]. One case report describes acute coronary artery thrombosis and vasospasm following capecitabine and oxaliplatin treatment for colon cancer [10]. These reports further emphasize the prothrombotic potential of this combination chemotherapy regimen.

## Conclusions

This case report highlights the importance of awareness of potential thrombotic complications in patients undergoing chemotherapy. The reported cases and available literature suggest an association between arterial thrombosis and a combination of capecitabine and oxaliplatin chemotherapy. In this case, the incidental discovery of an aortic thrombus in this patient with stage IIIA colon cancer who had recently started adjuvant chemotherapy with capecitabine and oxaliplatin further underscores the need for close monitoring of patients on these regimens. Further studies are needed to investigate the mechanism between chemotherapy regimens and thrombotic events in cancer patients. Clinicians should be aware of this potential complication and should consider appropriate prophylactic measures in patients at high risk for thrombosis.
